# Varus shearing force is a main injury mechanism of pediatric trampoline-related injury in addition to compressive axial loading

**DOI:** 10.1371/journal.pone.0217863

**Published:** 2019-06-05

**Authors:** Keong-Hwan Kim, Han-Soo Kim, Michael Seungcheol Kang, Soo-Sung Park

**Affiliations:** 1 Department of Orthopaedic Surgery, Asan Medical Center Children's Hospital, University of Ulsan College of Medicine, Seoul, Republic of Korea; 2 Department of Orthopaedic Surgery, Kangwon National University Hospital, Chuncheon-si, Gangwon-do, Republic of Korea; 3 Department of Orthopaedic Surgery, Seoul National University Hospital, Seoul, Republic of Korea; Istituto Di Ricerche Farmacologiche Mario Negri, ITALY

## Abstract

**Background:**

Many case studies have been published about trampoline-related injury (TRI); however, a comparative study could allow a more specific analysis of the characteristics of TRI, and enable more differentiated approaches to prevent such injuries. We investigated the injury mechanism of TRI in children compared with other pediatric trauma.

**Methods:**

Of 35,653 children (age 0–18 years) who visited the pediatric emergency department after traumatic injuries from January 2011 to June 2017, 372 patients with TRI (TRI group) were retrospectively identified. Among the remaining 35,281 patients with other trauma (non-TRI group), 372 were 1:1 matched to the TRI group according to sex, age, injured body part, and body weight (matched-control group). The patients’ data, injured site, and injury patterns were compared between the groups.

**Results:**

The most frequently injured body part was the knee/lower leg in the TRI group and the head in the non-TRI group. The most frequent injury types were fractures in the TRI group and open wounds in the non-TRI group. In the comparison between the TRI and matched-control groups, the most common lower-extremity fractures were proximal tibial fractures with varus angulation in the TRI group and tibial shaft spiral fractures in the matched-control group. For the upper extremities, the risk of lateral condylar humeral fracture was higher in the TRI group. The TRI group presented more physeal involvements.

**Conclusions:**

The risks of varus stress injury (proximal tibial fracture with varus angulation in lower extremity and lateral condylar humeral fracture in upper extremity) were higher in the TRI group than in matched-control group. Thus, varus shearing force seems to be an important injury mechanism in TRI in addition to compressive force. This varus force may increase the risk of physeal injury by generating additional shear force on the physis.

## Introduction

The incidence of trampoline-related injuries (TRIs) in children has increased over decades [1–4]. The patterns of TRI have been described in previous studies [5, 6], including a large-scale study using a national electronic surveillance system [7]. Consequently, several injury patterns of TRI and recommendations to prevent such injuries in relation to the design of trampolines and the behavior of the jumpers have been introduced and updated [7, 8].

However, studies to date have focused on the characteristics and patterns of TRI alone. There is a lack of comparative studies between TRI and other pediatric traumatic injuries. Although an epidemiologic study with a large sample size is meaningful, a comparative study has its own advantages in revealing the nature of the injury mechanism and might enable more differentiated approaches to prevent TRI.

To perform such comparative studies, it is necessary to appropriately control for bias. However, this is especially difficult in children because physical trauma differs greatly according to age [9], obesity [10], and sex [11]. Additionally, there are also significant differences in the commonly injured body part according to the activity type [12].

Excessive axial loading during landing (i.e., compressive force) is a well-known injury mechanism of TRI [7, 13], and we hypothesized that there is an additional force other than pure compression in this kind of injury. The purpose of the present study was to investigate the injury mechanism of TRI in detail. To achieve this purpose, we analyzed the characteristics of TRI in children compared with those of other pediatric physical trauma.

## Materials and methods

The present study protocol was reviewed and approved by the Institutional Review Board of Asan Medical Center (approval No. 2017–0384). Informed consent was waived because the data were gathered retrospectively and analyzed anonymously.

### Patient selection

From January 2011 to June 2017, a total of 36,799 patients aged ≤18 years visited the pediatric emergency department (PED) of our institute after a traumatic injury. Of them, the patients with any missing data (n = 1146) were excluded, and the other 35,653 patients had all the required data including sex and age, diagnostic code, and body weight at the PED visit. The electronic medical charts were searched using words related to TRI, and 385 patients were selected. All of the searched medical records were individually reviewed, and 372 patients whose trauma was truly related to trampoline use were identified. These 372 patients with TRI were classified as the TRI group, whereas the remaining 35,281 patients were considered the non-TRI group. Among the non-TRI group, 372 patients were matched at a 1:1 ratio to the TRI group individually, in terms of sex, age, injured body part, and body weight. If there were two or more candidates with entirely the same matching variables, the patient who visited the PED at a date nearest to that of the patient with TRI was selected. Those 372 matched patients were classified as the matched-control group. The injured body part was determined using the diagnostic code assigned at the PED visit according to the International Classification of Diseases 10th revision (ICD-10). The institutional review board of our institute approved this study.

### Investigated variables

Sex, age, injured body part, injury type according to the ICD-10 diagnostic code, and body weight were investigated in both the TRI (n = 372) and non-TRI (n = 35,281) groups.

For the TRI (n = 372) and matched-control (n = 372) groups, all medical charts and radiological evaluations were individually reviewed, and detailed data about the injured body part and diagnosis, severity of injury, and presence of physeal involvement were investigated. The severity of injury was classified as mild, moderate, and severe. Cases that needed a certain procedure under general anesthesia were considered severe. Mild cases were considered those that needed no further treatment other than short-term immobilization or simple dressing, such as simple abrasion, sprain, or contusion. The remaining cases were considered moderate. For patients with fractures, all radiographs taken at the PED and during follow-up visits were reviewed. The presence of a deformity was identified if there was an angular deformity >5° in any direction when compared with the contralateral side at post-trauma 1 year, or if surgical correction was needed during the follow-up.

### Statistical analysis

Statistical analyses were performed using SPSS software (version 21; IBM Co., Armonk, NY, USA). To compare the proportion of each variable, the chi-square test or Fisher’s exact test was used. A statistically significant difference was considered present at P < 0.05.

## Results

The demographic data of the TRI and non-TRI groups are shown in Table 1. Most injuries occurred in the head in the non-TRI group; however, the TRI group showed more frequent injuries in the extremities, especially the shoulder/upper arm, knee/lower leg, and ankle/foot. The most common types of injury were open wounds in the non-TRI group and fractures in the TRI group. There was no case with significant multiple injuries in the TRI and matched-control groups.

**Table 1:** Comparisons between the TRI and non-TRI groups.

	TRI group (n = 372)	Non-TRI group (n = 35,281)	P value
Sex			<0.001
Male	195 (52.4%)	12,979 (36.8%)	
Female	177 (47.6%)	22,302 (63.2%)	
Age (years)	5.2 ± 3.2 (0–17)	4.6 ± 4.3 (0–18)	<0.001
0–1	34 (9.1%)	10,240 (34.2%)	
2–3	104 (28.0%)	9070 (25.7%)	
4–5	79 (21.2%)	5222 (14.8%)	
6–7	69 (18.5%)	3148 (8.9%)	
8–9	37 (9.9%)	2190 (6.2%)	
10–11	35 (9.4%)	1624 (4.6%)	
12–13	11 (3.0%)	1648 (4.7%)	
14–15	2 (0.5%)	1377 (3.9%)	
16–18	1 (0.3%)	732 (2.2%)	
Diagnostic code (injured body part)			
S0 (head)	73 (19.6%)	**23,911 (67.8%)**	<0.001
S1 (neck)	9 (2.4%)	395 (1.1%)	0.018
S2 (chest)	4 (1.1%)	177 (0.5%)	0.122
S3 (abdomen/low back/pelvis)	2 (0.5%)	683 (1.9%)	0.054
S4 (shoulder/upper arm)	**48 (12.9%)**	1134 (3.2%)	<0.001
S5 (elbow/forearm)	42 (11.0%)	3371 (9.6%)	0.258
S6 (wrist/hand)	11 (3.0%)	2415 (6.8%)	0.003
S7 (hip/buttock/thigh)	12 (3.2%)	204 (0.6%)	<0.001
S8 (knee/lower leg)	**117 (31.5%)**	1303 (3.7%)	<0.001
S9 (ankle/foot)	**55 (14.8%)**	1688 (4.8%)	<0.001
Diagnostic code (type of injury)*			
0 (Superficial wound)	66 (17.7%)	10,203 (28.9%)	<0.001
1 (Open wound)	39 (10.5%)	**12,410 (35.2%)**	<0.001
2 (Fracture)	**166 (44.6%)**	3982 (11.3%)	<0.001
3 (Dislocation/strain/sprain)	88 (23.7%)	3859 (10.9%)	<0.001
4 (Nerve injury)	0	13 (0.0%)	1.000
5 (Vessel injury)	0	347 (1.0%)	0.056
6 (Muscle/tendon injury)	8 (2.2%)	3793 (10.8%)	<0.001
7 (Crushing injury)	0	40 (0.1%)	1.000
8 (Traumatic amputation)	0	5 (0.0%)	1.000
9 (Unknown)	5 (1.3%)	629 (1.8%)	0.693
Body weight (kg)	22.1 ± 10.7 (9.0–69.0)	21.4 ± 15.6 (1.2–124.4)	0.236
	cf. Matched-control group: 22.0 ± 10.5 (9.1–58.0)		

*The diagnostic codes used were assigned at the pediatric emergency department (PED). Thus, the final diagnosis, which was confirmed after follow-up visits to the clinic, may be mismatched to the assigned diagnostic code at the PED.

TRI, trampoline-related injury.

### Comparison between the TRI and matched-control groups: Diagnosis

In the matched-control group, the causes of injuries were slipping down (n = 96), direct contact (n = 93), falling (n = 56), traffic accident (n = 40), penetration (n = 13), and others (n = 74). Table 2 presents the comparisons of laterality and diagnosis. Even after matching, the risk of fractures was still higher in the TRI group (bold). Table 3 presents the frequencies of diagnosis according to injured sites. There was no significant difference in injury types when the injured site was the head or trunk (P = 0.816). However, the injury types in the upper (P = 0.003) and lower (P < 0.001) extremities were significantly different between the groups.

**Table 2:** Comparisons between the TRI and matched-control groups: laterality and diagnosis.

	TRI group (n = 372)	Matched-control group (n = 372)	P value
Laterality			0.473
Both or NA	74 (19.9%)	91 (24.5%)	
Right	157 (42.2%)	150 (40.3%)	
Left	138 (37.1%)	127 (34.1%)	
Missing data	3 (0.8%)	4 (1.1%)	
Diagnosis			
Fracture	**170 (45.7%)**	114 (30.6%)	<0.001*
Dislocation	2 (0.5%)	0	0.499
Contusion/sprain	137 (36.8%)	139 (37.4%)	0.879
Laceration	37 (9.9%)	63 (16.9%)	0.005*
Abrasion	9 (2.4%)	27 (7.3%)	0.002*
Concussion	7 (1.9%)	7 (1.9%)	1.000
Tooth injury	5 (1.3%)	3 (0.8%)	0.725
Pulled elbow	4 (1.1%)	18 (4.8%)	0.004*
Hemotympanum	1 (0.3%)	0	1.000
Dog bite	0	1 (0.3%)	1.000

*P < 0.05.

TRI, trampoline-related injury; NA, not applicable.

**Table 3:** Comparisons between the TRI and matched-control groups: Diagnosis according to injured sites.

	TRI group (n = 372)	Matched-control group (n = 372)	P value
*Head/trunk*	*Subtotal n = 88*	*Subtotal n = 88*	0.816
Laceration	37 (42.0%)	39 (44.3%)	
Contusion/sprain	26 (29.5%)	31 (35.2%)	
•Head	12	18	
Abrasion	8 (9.1%)	6 (6.8%)	
Concussion	7 (8.0%)	7 (8.0%)	
Tooth injury	5 (5.7%)	3 (3.4%)	
Fracture	4 (4.5%)	2 (2.3%)	
Hemotympanum	1 (1.1%)	0	
*Upper extremity*	*Subtotal n = 100*	*Subtotal n = 100*	0.003*
Fracture	80 (80.0%)	59 (59.0%)	
Around the elbow	56	25	
•Supracondylar humerus	24	18	
•Lateral condylar humerus	**19**	**4**	
•Distal radius/ulna	4	12	
•Clavicle shaft	2	13	
Contusion/sprain	15 (15.0%)	16 (16.0%)	
Pulled elbow	4 (4.0%)	18 (18.0%)	
Dislocation	1 (1.0%)	0	
Abrasion	0	3 (3.0%)	
Laceration	0	3 (3.0%)	
Dog bite	0	1 (1.0%)	
*Lower extremity*	*Subtotal n = 184*	*Subtotal n = 184*	<0.001*
Contusion/sprain	96 (52.2%)	92 (50.0%)	
•Ankle	46	21	
•Knee	32	30	
•Lower leg	14	15	
•Foot	6	19	
Fracture	86 (46.7%)	53 (28.8%)	
Around the knee	56	10	
•Proximal tibia	**43**†	6‡	
With varus angulation**	19	1	
Neutral	20	2	
With valgus angulation**	4	3	
•Distal femur	11†	3‡	
•Tibial shaft	1	**24**	
•Malleolus (medial/lateral/bilateral)	16	3	
Abrasion	1 (0.5%)	18 (9.8%)	
Dislocation	1 (0.5%)	0	
Laceration	0	21 (11.4%)	

*P < 0.05.

**If there was a ≥2° angulation compared with the contralateral uninjured side.

†A total of 97.7% (42/43) proximal tibial fractures and 90.1% (10/11) distal femoral fractures occurred in children aged ≤6 years.

‡All patients with proximal tibial and distal femoral fractures in the matched-control group were aged ≤6 years.

•Only specific diagnoses with >10 cases were additionally presented in the table.

TRI, trampoline-related injury; N/A, not applicable.

For the upper extremity, fractures around the elbow were more frequent in the TRI group [56.0% (56/100) vs. 25.0% (25/100)]. For the specific types of fracture, the frequencies of supracondylar humeral fractures (the most common fracture type) were not different between the groups [30.0% (24/80) in the TRI group and 30.5% (18/59) in the matched-control group, P = 0.949]; however, the frequencies of lateral condylar humeral fractures were significantly higher in the TRI group (23.8%, 19/80) than in the matched-control group (6.8%, 4/59) (P = 0.010, bold in the middle row of Table 3).

For the lower extremity, the most common fracture types were proximal tibial fractures in the TRI group and tibial shaft fractures in the matched-control group (bold in the lower row of Table 3). To assess the direction of the injury force, the angulation of proximal tibial fractures was additionally investigated. Half of the matched-control group (3 of 6, 50%) presented valgus angulation of the proximal tibia, but only 9.3% (4 of 43) presented valgus angulation in TRI group (P = 0.031).

All but one fracture in the distal femur and proximal tibia were transverse or short oblique metaphyseal fractures, mostly with cortical buckling or mild angulation. The one exception was a proximal tibial fracture in a 10-year-old girl who was injured with a direct blow to her knee while falling on the ground in a kneeling position. This patient had physeal separation of the proximal tibia without metaphyseal extension. On the contrary, the tibial shaft fractures, which mostly occurred in the matched-control group, consisted of long oblique fractures in two cases and spiral fractures in the others.

### Comparisons between the TRI and matched-control groups: Severity

The matched-control group presented a higher frequency of mild injuries than the TRI group (P < 0.001), and the TRI group presented more physeal injuries than the matched-control group (P < 0.001) (Table 4).

**Table 4:** Comparisons between the TRI and matched-control groups: Severity.

	TRI group (n = 372)	Matched-control group (n = 372)	P value
Severity*			<0.001
Severe	34 (9.1%)	30 (8.1%)	
Moderate	145 (39.0%)	90 (24.2%)	
Mild	193 (51.9%)	252 (67.7%)	
Physeal injury			<0.001
Present	85 (22.8%)	28 (7.5%)	
Deformity (+)†	4 (4.7%)	0	
Deformity (-)†	81 (95.3%)	28 (100%)	
Absent	287 (77.2%)	344 (92.5%)	

*Severe: surgical treatments were needed to treat the patients. Mild: trauma that needed no further treatment other than short-term immobilization or simple dressing, such as simple abrasion, sprain, contusion. Moderate: all others.

†Deformity was defined as present if there was an angular deformity >5° in any direction when compared with contralateral side at post-trauma 1 year, or if surgical correction was needed during the follow-up.

TRI, trampoline-related injury.

## Discussion

We investigated the characteristics of children with TRI (TRI group) and compared them with those of children with a traumatic injury other than TRI (non-TRI group). The most frequent injured body part was the head in the non-TRI group and the knee/lower leg in the TRI group. The most frequent kinds of injuries were open wounds in the non-TRI group and fractures in the TRI group.

For more accurate comparisons, the TRI group was matched at a 1:1 ratio according to age [9], body weight [10], sex [11], and injured body part [12] (matched-control group). Even in the matching comparisons, fracture was still more common in the TRI group than in the matched-control group. The most common fractures were proximal tibial fractures in the TRI group and tibial shaft fractures in the matched-control group (Table 3). For proximal tibial fractures, the TRI group presented more neutral or varus angulation compared with the matched-control group, which presented a more valgus angulation. For elbow fracture, which was the most common injury in the upper extremity, the risk of lateral condylar humeral fractures was higher in the TRI group than in the matched-control group. Transverse or short oblique metaphyseal fracture was the typical fracture pattern of the lower extremity in the TRI group. The TRI group tended to have more physeal involvements.

**Fig 1:**
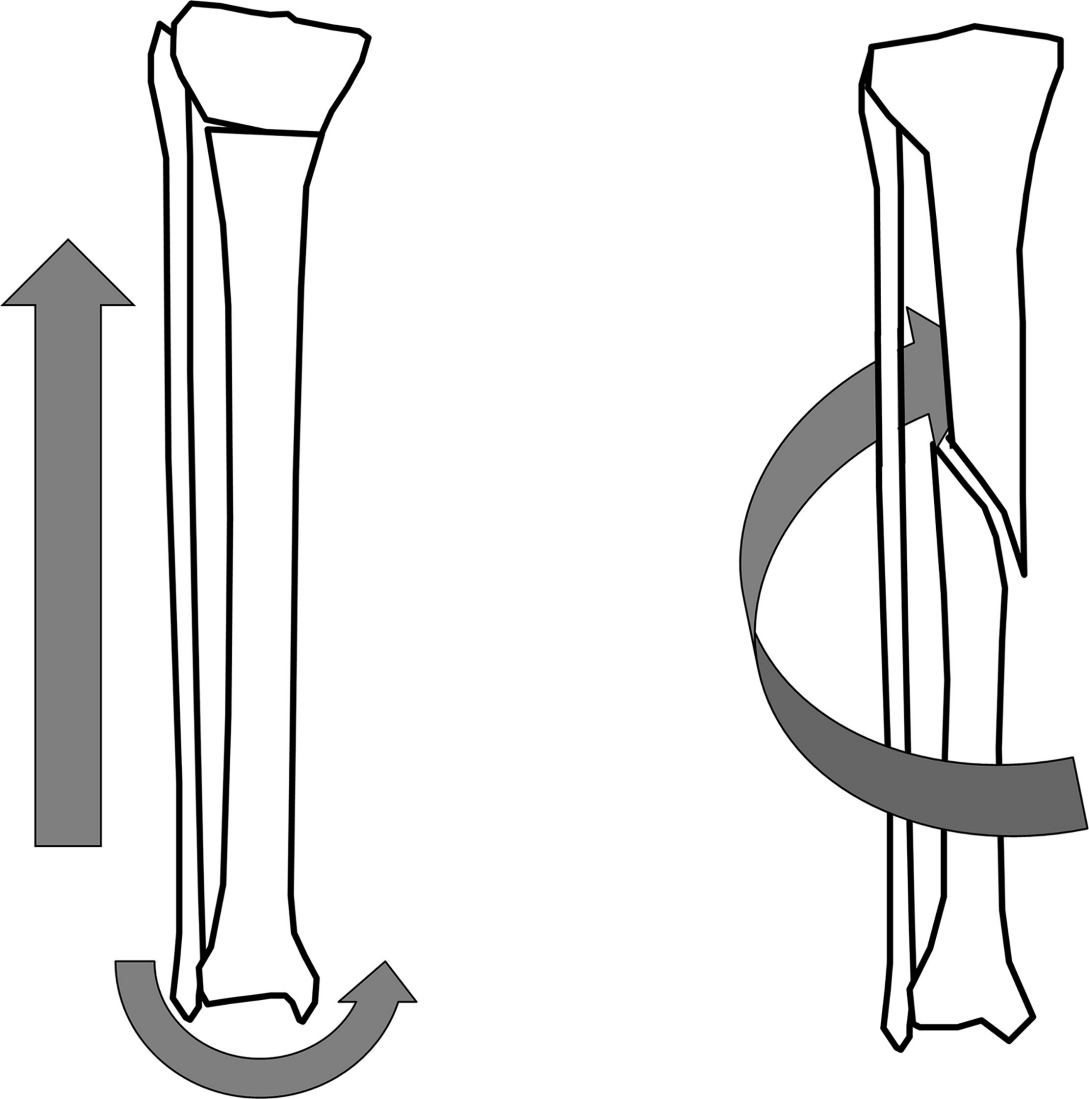
Common tibial fracture patterns in the trampoline-related injury (TRI) and matched-control groups. For the TRI group, the most common tibial fracture pattern (42 of 184 lower-extremity injuries) was proximal tibial fracture with a transverse or short oblique fracture line (left), and most of them presented neutral or varus angulation. In contrast, for the matched-control group, cases with proximal tibial fracture were relatively rare (only 6 of 184 lower-extremity injuries) and half of the patients with a proximal tibial fracture presented valgus angulation. Therefore, compression with varus shearing force seems to be the main injury mechanism in TRI. On the other hand, tibial shaft fractures, which mostly occurred in the matched-control group, presented as long oblique or spiral fractures, which indicate a torsional injury mechanism (right).

**Fig 2:**
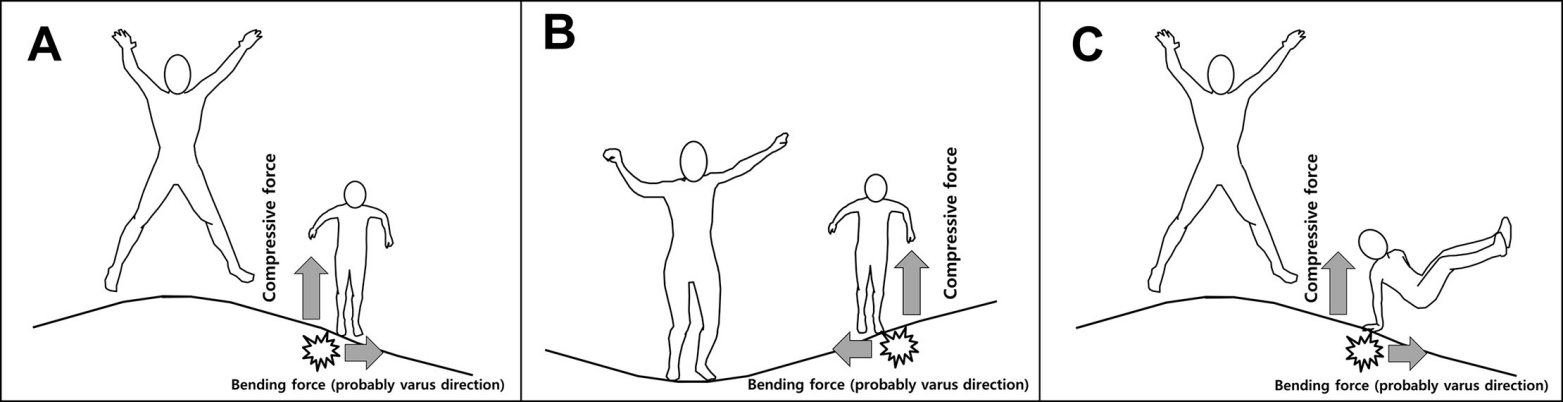
Varus shearing force in trampoline-related injury. (A) It is known that when multiple children are using a trampoline at the same time, the smaller children receive excessive axial loading during landing due to the upward recoil on the mat caused by a heavier jumper [7, 13]. However, when considering the presence of cortical buckling and angulation, a somewhat additional varus shearing force seems to be combined with the compressive force (gray arrows). Although more studies are needed, we postulate that this varus force originates from the inclined mat. (B) Even when a smaller jumper lands on the trampoline simultaneously with a heavier jumper, the inclined mat can cause varus stress. (C) This assumption can also be supported by the incidence of elbow fractures. Lateral condylar humeral fractures, which are associated with hyperextension with varus stress in injuries involving falling on a stretched arm, were relatively more frequent in the group with trampoline-related injuries (Table 3).

We attempted to find differences in the main injury mechanism. In both the TRI and matched-control groups, the tibia was the most commonly fractured site. However, the detailed lesion and fracture patterns were different between the groups. Proximal tibial transverse fractures with varus deformity were common in the TRI group, whereas tibial shaft spiral fractures were the most common injuries in the non-TRI group (Fig 1). Therefore, we postulate that tibial fractures resulting from traumatic injury other than TRI are often caused by torsional forces, but compression with bending forces seem to be predominant in TRI. In particular, the bending force is likely to act as a varus force. Jumping on a trampoline with a heavier child can cause an upward recoil that leads to excessive axial loading in the smaller child during landing, and this is a well-reported injury mechanism of TRI [7, 13]. We believe that such compression force would be still the main force, but some degree of varus force also seems to be apparent. This hypothesis can also be supported by the incidence of elbow fracture subtypes. Although the risk of supracondylar humeral fracture was similar, the risk of lateral condylar fracture was higher in the TRI group than in the matched-control group (Table 3, bold in the middle row). The typical cause of distal humeral fractures in children is a fall onto an outstretched elbow [14]. When a child falls onto an outstretched arm, the injury mechanism of hyperextension with vertical stress is associated with supracondylar humeral fracture, that of hyperextension with valgus stress is associated with radial head/neck fracture, and that of hyperextension with varus stress is associated with lateral condylar fracture [14, 15]. Therefore, the higher risk of lateral condylar humeral fracture means that some varus forces seem to be applied in addition to the compressive force in TRI. Conclusively, although simple compressive force (upper extremity) and torsional force (lower extremity) seem to be the main injury mechanisms in non-TRI, compression and additional varus shearing force might be the main injury mechanisms in TRI (Fig 2).

**Fig 3:**
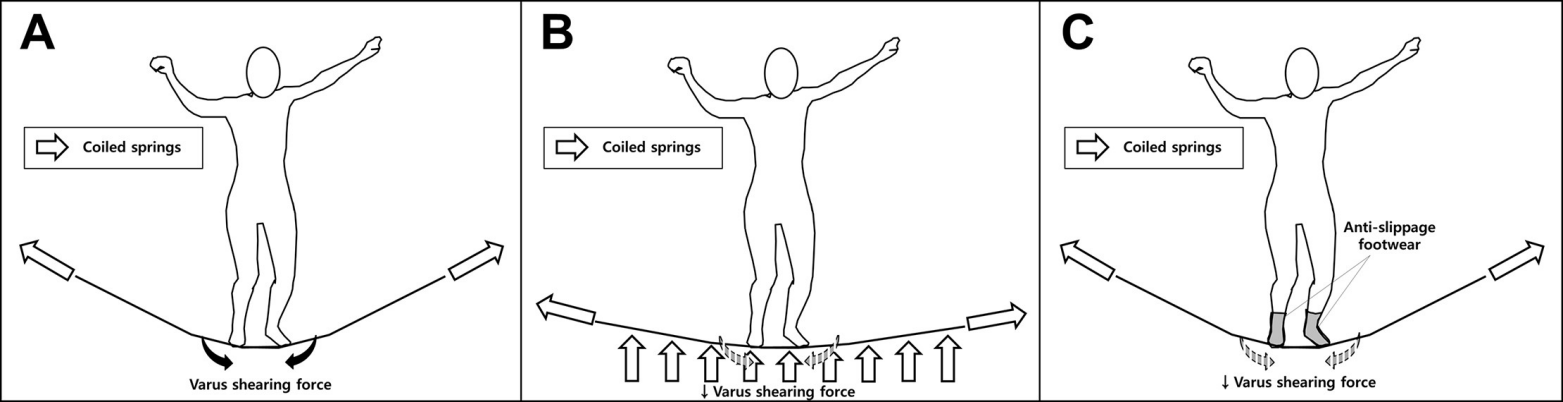
Suggested preventive measures for trampoline-related injuries (TRIs). Under the assumption that the varus shearing force plays an important role in the occurrence of TRI, altering the design of trampolines or using specially designed footwear may contribute to preventing TRI or reducing TRI-related physeal injuries. (A) The trampoline consists of a taut and strong mat that is connected to a peripheral steel frame with many coiled springs. Because of this structure, when a child lands on the mat, the mat becomes inclined to the center, generating varus force. (B) Therefore, it can be postulated that the varus shearing force can be reduced by using additional coiled springs that are vertically positioned. (C) With respect to the varus shearing force caused by slippage of the foot and the inclined mat, wearing of anti-slippage footwear during trampoline use might also prevent injury.

There has been a relative lack of studies about trampoline-related physeal injury and the development of deformity. McDermott et al [6] reported that 15% of TRIs are accompanied by physeal involvement. None of the patients developed deformity after “trampoline fracture” of the proximal tibia in two previous studies [13, 16]. Blumetti et al [17] reported two cases of varus deformity that occurred after severe medial malleolar physeal injury due to trampoline activity. In our study, although the risk of residual deformity was low (4 of 85 cases presented a residual angular deformity >5° compared with contralateral side at 1 year after trauma among trampoline-related physeal injuries; Table 4), the incidence of physeal injury in the trampoline group was higher than in the control group. We believe that physeal injury may occur more frequently in TRI because it accompanies the varus bending force that can generate shearing force on the growth plate. Despite the low risk of persistent deformity, the possibility of a physeal injury should be a concern and susceptible patients should be followed up serially. Although more studies are certainly needed, some preventive measures can be carefully suggested under the assumption that this varus force is also responsible for TRI (Fig 3).

There are some considerations in interpreting the results of the present study. First, we selected 372 patients with TRI from among about 35,000 patients with traumatic injury who visited the PED of our institute. Although this study has one of the largest sample sizes among the single-center studies [2, 6, 16, 18, 19], there remains a sample size problem. For example, the tibial shaft fracture of the matched-control group was either a spiral fracture or a long oblique fracture. However, this does not mean that no other types of fracture, such as transverse fractures, occur from general physical trauma. It would be more appropriate to interpret that the rate of occurrence of other fractures is relatively low and that it was not revealed with the current sample size. Second, sex, age, injured body part according to diagnostic code, and body weight were used for matching the control group. Because the characteristics of pediatric physical trauma widely differ depending on these individual features, it is necessary to use matching comparisons to investigate the mechanism of a specific injury. However, further research on the matching method is certainly needed for a more accurate comparison. In contrast, the characteristics of the matched-control group should not be misunderstood as the characteristics of the non-TRI group. As the matched-control group comprised only those patients selected to match TRI group, they cannot represent the entire non-TRI group. Additionally, with respect to matching, when a child visits the PED at our institute, the weight is checked and recorded routinely, but not the height. Therefore, although a matching comparison with body mass index would be more effective in removing bias [10], only body weight was used for matching instead of body mass index. Third, there is another consideration with the use of the diagnostic code for matching of the injured body part. For example, proximal tibial and distal femoral fractures are generally considered fractures around the knee. However, when the diagnostic code is used, the body parts were classified as the tibia and femur, respectively, because the diagnostic code is based mainly on the injured bone. These mismatches should be kept in mind when interpreting our results because they might cause misconceptions. Fourth, there are many classifications of injuries, but most of them were established for patients requiring critical care. Such classifications do not fit well with our study because most of our cases involved relatively mild injuries that did not require critical care. Thus, we used our own criteria to classify the injury severity. Fifth, there was no case with multiple injuries in the TRI and matched-control groups, but there were cases with multiple injuries in the non-TRI group. The results presented for the non-TRI group were based solely on the diagnostic code that was registered as the main diagnosis.

In conclusion, we investigated the characteristics of children with TRI (TRI group) and compared them with those of children with traumatic injury other than TRI (non-TRI group) and with a matched-control group. Simple compressive force (upper extremity) and torsional force (lower extremity) seem to be the main injury mechanism in the matched-control group; however, compression with additional varus force seems to be the main injury mechanism in TRI group. Children with TRI appear to have more frequent physeal injuries than their matched-control counterparts. The additional varus force may increase the risk of physeal injury by generating additional shearing force on the physis.

## Supporting information

S1 File Data of the TRI and matched-control groups. (SAV)
